# A Case of Pseudogout Causing Thoracic Myelopathy

**DOI:** 10.7759/cureus.30258

**Published:** 2022-10-13

**Authors:** Emilee A Carpenter, Zaid Siddique, Ola El-Zammar, Adriana May, Kavya Mirchia

**Affiliations:** 1 Radiology, State University of New York Upstate Medical University, Syracuse, USA; 2 Pathology, State University of New York Upstate Medical University, Syracuse, USA; 3 Neuroradiology, State University of New York Upstate Medical University, Syracuse, USA

**Keywords:** calcium pyrophosphate deposition disease, myelopathy, pathology of pseudogout, pseudogout imaging, thoracic spine lesion

## Abstract

Calcium pyrophosphate deposition disease is not an uncommon cause of polyarthritis, especially in the elderly. This disease typically affects the appendicular skeleton but may rarely affect the axial skeleton as well. When the axial skeleton is involved, it can lead to numerous neurological signs and can be disabling. We describe a case in which a 68-year-old male presented with on-and-off myelopathy and was thought to have chronic inflammatory demyelinating polyneuropathy. Magnetic resonance imaging of the spine suggested an inflammatory or infectious lesion at the thoracic level. However, after a surgical biopsy, pathologists concluded that calcium pyrophosphate deposition, or pseudogout, was the cause of this patient’s neurological symptoms. Pseudogout in the spine, especially the thoracic spine, is exceptionally rare. There are very few additional cases reported. In this report, we review the current literature on existing similar cases, radiological findings, risk factors, and treatments for this condition in hopes of increasing knowledge and awareness of this rare differential.

## Introduction

Calcium pyrophosphate deposition (CPPD) disease, otherwise known as pseudogout, occurs when calcium pyrophosphate crystals deposit in the pericellular matrix of cartilage, often affecting the appendicular skeleton [1]. It occurs most commonly in patients older than 65 years and its prevalence increases with age [2]. It is estimated that the prevalence of CPPD is between 7% and 10% in individuals over the age of 60 [3].

CPPD tends to affect predisposed joints such as those affected by inflammatory conditions (i.e. rheumatoid arthritis), degeneration, or trauma. While the knee is the most common location, the wrists, ankles, elbows, toes, and shoulders are often affected as well [4]. Rarely, CPPD can occur in the spinal column.

We describe a rare case in which a patient has experienced neurologic deficits secondary to CPPD in the thoracic spine. There are very few identified cases such as this, as crystal deposition in this location is rare due to unclear mechanisms. We hope to increase awareness about CPPD in this location, as it can mimic other diseases on imaging.

## Case presentation

A 68-year-old male presented to the hospital due to worsening bilateral leg paresthesias, weakness, and multiple falls. These symptoms were preceded seven months prior with paresthesias and progressive proximal lower limb weakness one month after an upper respiratory infection. The patient was evaluated at that time with electromyography (EMG), which suggested demyelinating neuropathy consistent with acute inflammatory demyelinating polyneuropathy (AIDP) or multifocal motor neuropathy. He was treated with a three-day course of intravenous immunoglobulin (IVIG). His symptoms including leg strength seemed to improve over the next few months.

However, more recently, the symptoms returned and were progressively worsening. The patient’s relevant medical history includes alcoholism, hyperlipidemia, and seronegative rheumatoid arthritis (RA). Neurologic examination was normal except for decreased patellar and Achilles reflexes bilaterally and decreased sensation to pinprick along the dorsal surface of the feet bilaterally. The plantar response was flexor bilaterally.

Relevant labs include elevated creatinine kinase (260, normal range 20-200), mean corpuscular volume (100.4, normal range 80-96), and cerebral spinal fluid (CSF) protein (62, normal range 15-45). The patient presented with no infectious signs and all other laboratory studies showed no abnormalities. The patient was started an IVIG infusion for concerns about AIDP progressing to chronic inflammatory demyelinating polyneuropathy (CIDP). MRI of the spine was also ordered.

**Figure 1:**
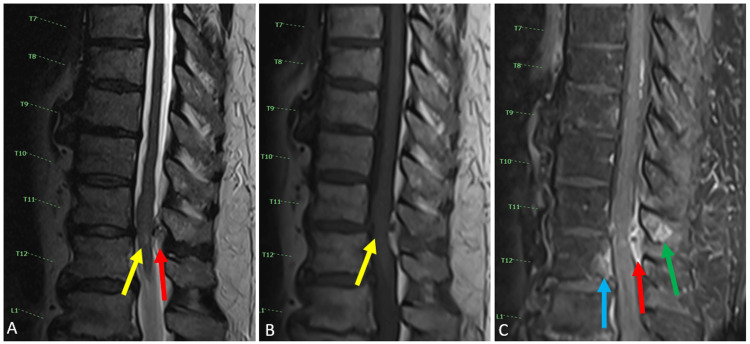
Sagittal MRI of the Thoracic Spine With and Without Contrast Sagittal MRI of the thoracic spine with T2 image (A), T1 image (B), and T1 post-contrast fat saturation image (C). Figure A demonstrates the expansion of the conus medullaris with an abnormal focus of T2 hyperintense signal (yellow arrow) with severe spinal canal stenosis caused by a heterogenous posterior epidural lesion (red arrow). Figure B demonstrates severe spinal canal stenosis (yellow arrow). Figure C demonstrates abnormal enhancement of the posterior epidural lesion (red arrow), which causes severe spinal canal stenosis. There is an abnormal enhancement of the posterior cortex of the T12 vertebral body (blue arrow) and an enhancement of the T11 spinous process (green arrow).

**Figure 2:**
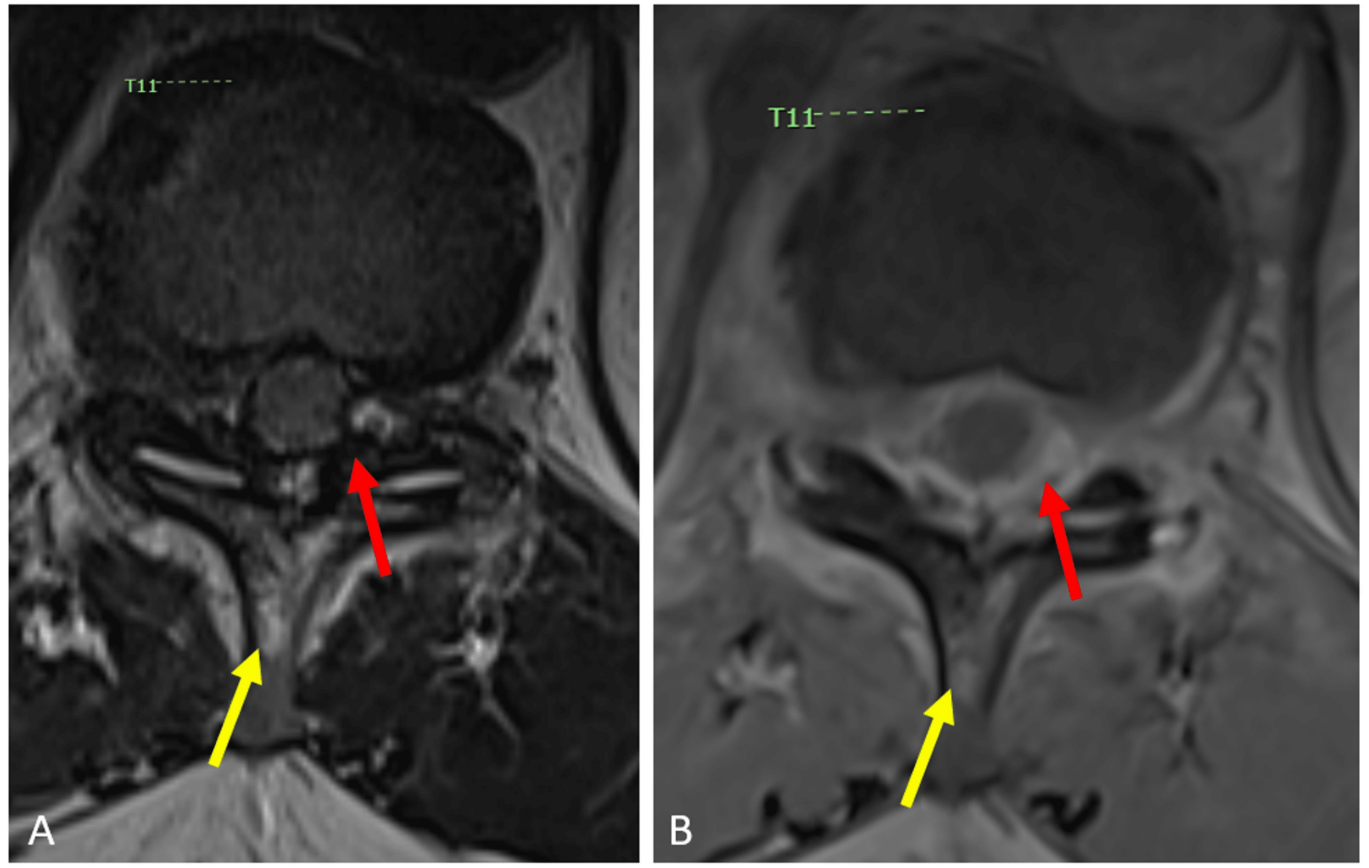
Axial MRI of the Thoracic Spine With and Without Contrast Axial MRI images with T2 (A) and T1 post-contrast fat saturation (B) of the thoracic spine at the level of T11. There is a heterogenous collection that demonstrates avid enhancement in the posterior epidural space causing severe spinal canal stenosis with effacement of the thecal sac (red arrow). There is a T2 hyperintense signal with corresponding contrast enhancement within the T11 spinous process (yellow arrow).

MRI with and without contrast showed an abnormal enhancing extradural collection/lesion as well as enhancement of the adjacent T11 and T12 vertebral bodies (Figures 1, 2). The concern at the time was abscess or phlegmon formation with osteomyelitis. There was also an expansion of the cord, severe central canal stenosis, and compression of the conus medullaris at this level. Thickening and enhancement in the conus medullaris suggested inflammatory changes. Other non-pertinent findings included varying levels of neural foraminal stenosis including areas of severe neuroforaminal stenosis within the cervical spine.

**Figure 3:**
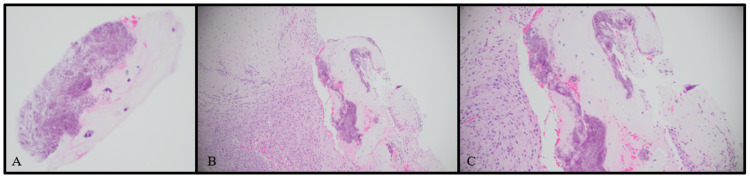
Hemosiderin and Eosin (H&E) Stain of the Surgical Specimens Collected at the T11 Level H&E stain of the T11 spinous process biopsy (A), H&E stain of the T11 ligamentum flavum biopsy (B), H&E stain of the epidural lesion biopsy (C). Figure A demonstrates fragments of benign bone with focal marrow fibrosis and hemosiderin-laden macrophages and cartilage with degenerative changes. Figure B demonstrates fragments of cartilage with degenerative changes and dense fibrous tissue with focal granulation tissue formation. Figure C demonstrates synovial-lined dense fibrous tissue with calcification and granulation tissue.

**Figure 4:**
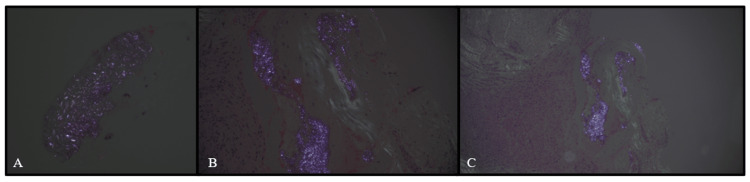
Surgical Specimens of the Thoracic Spine Subjected to Polarized Light T11 spinous process biopsy (A), T11 ligamentum flavum biopsy (B), and epidural lesion biopsy (C), all subject to polarized light. All figures demonstrate crystals with positive birefringence, consistent with pseudogout.

Neurosurgery performed a T11 to T12 laminectomy and biopsies were taken of the lesion. Grossly, these lesions appeared consistent with synovial cysts. IVIG infusion was continued for five days. Pathology results of the epidural lesion showed synovial-lined dense fibrous tissue with calcification, granulation tissue, and birefringent crystal deposition, consistent with pseudogout (Figures 3, 4).

The patient was discharged from the hospital to home several days later. However, on reexamination prior to discharge, patellar and Achilles reflexes and pinprick sensation were unchanged from the pre-surgical examination.

## Discussion

CPPD is an idiopathic condition, however, it has a predilection to affect patients with certain risk factors. Aging, previous joint injury, osteoarthritis, hyperparathyroidism, hypomagnesemia, hypothyroidism, and hemochromatosis are some of these risk factors [4,5]. Chronic cases of CPPD may mimic rheumatoid arthritis or osteoarthritis [6]. It is possible that this patient’s chronic seronegative RA may have been misdiagnosed. However, RA could have predisposed him to develop CPPD disease due to joint destruction, though the lack of involvement of the thoracic spine in RA would suggest its association is less so in this case.

Imaging modalities that are typically used to diagnose suspected CPPD are radiography and CT. Given that this patient was suspected to be progressing from AIDP to CIDP, MRI was ordered. MRI findings in CPPD are typically nonspecific, but may show erosions, synovitis, and marrow edema [7] and should be followed up with CT to distinguish infectious conditions or tumors from crystal arthropathies [8]. This patient’s MR study suggested an inflammatory or infectious process such as abscess or phlegmon formation. Other differentials that were considered included vascular congestion, cord ischemia, and myelitis.

When the spine is involved in CPPD, it most commonly affects the cervical region followed by the lumbar segments [9]. The thoracic spine is the least common location [10]. There also may be an increased prevalence of spinal CPPD in females, which makes this case even rarer [6]. Most often, the ligamentum flavum or periodontoid structures in the cervical region are involved in cases of spinal CPPD [11]. The latter of the two is also known as Crowded Dens syndrome [6].

There are a limited number of other cases of CPPD occurring in the spine. Similar cases have been described in Table 1. Given the rarity of this diagnosis, it was presumed that our patient had an abscess or phlegmon with a component of osteomyelitis. There is a case report in which a patient with lumbar spinal CPPD was assumed to have osteomyelitis due to increased MRI signal intensity after months of lower back pain and neurological symptoms [12]. An additional report discussed a case of CPPD that presented with elevated inflammatory markers and was initially treated for presumed septic arthritis of the spine [13]. Our patient did not present with any signs or symptoms of infection. There are a few cases of spinal CPPD reported after spinal injury post-trauma. For example, there are a few reported cases of trauma-induced CPPD of the lumbar spine [14]. Another describes cervical and thoracic CPPD five weeks status post lumbar fusion procedure [15]. Lastly, there was a case of a younger patient with no known risk factors for CPPD who was diagnosed with CPPD of the thoracic spine and treated with laminectomy [16]. Spinal CPPD is rare in itself, and our patient’s location of involvement makes this case even more unique.

**Table 1:** A Case of Pseudogout Causing Myelopathy Within the Thoracic Spine; Review of Literature

	Moon [6]	Miura [17]	Srinivasan [10]	Bridges [15]	Muthukumar [16]	Muthukumar [18]	Paolini [19]	Mutschler [20]	Our Case
Type of report (number of patients)	case series (1 thoracic case)	case report	case report	case report	case report	case series (4 thoracic)	case series (2)	case report	case report
Patient Gender	M	M	M	F	F	3F / 1M	F	F	M
Age	66	78	72	66	45	45-60	36 / 43	41	68
Relevant History	History of L1-L3 posterior spinal instrumented fusion 8 years prior to onset	CAD, HTN		History of lumbar laminectomies and fusion 5 weeks prior to onset		No systemic features of CPPD		Infiltrating breast carcinoma	Seronegative RA
Spinal Level	T12/L1 facet	T11-T12	T9-T10	C7-T1 facet joints, T1-T2, T9-T12 intervertebral discs, T9-T12 ventral epidural space	T8-T11	T8-T9, T10-T11, T10-T12, T7-T8,	T9-T10 both cases	T9-T10	T11
Significant symptoms	Back pain, radicular pain in one lower extremity	Bilateral femoral numbness, gait disturbances, urinary symptoms	Left-sided chest pain	Worsening lower back pain, bilateral paresthesias	Lower extremity stiffness, weakness, sensory loss below T10	All patients except 1 presented with an insidious onset of myelopathy, the last patient had paraplegia after trauma	1 month of left-sided thoracic pain/left hemithorax burning pain for 6 months	Mid-back pain	Bilateral paresthesias, lower limb weakness. URI months prior.
MRI findings	Non-contrast imaging showed a large facet cyst with lateral recess stenosis and spinal cord compression	Posterior cord compression, hypodense mass, and intramedullary high-intensity signal on T2 weighted images	T9-T10 disc herniation with foraminal stenosis and T10 nerve root compression on T2 weighted images	Contrast enhancement within the C7–T1 facet joints as well as the T1–2 and T9–12 intervertebral discs. There was also ventral epidural enhancement from T9 to T12	T1-weighted images of the lower thoracic spine showing a round, hypointense lesion located posteriorly in the lower thoracic region, indenting the cord	Ossified ligamentum flavum showing up as hypodense signals on both T1 and T2 causing compression.	T9-T10 lateral disc herniation, hypodense on T1 and T2 weighted images / Hypodense nodular lesion effacing the neural foramen at T9-10	sagittal T1 images showed a left posteroinferior corner of T9 with low signal intensity. Sagittal T1 and T2 images at the T9-T10 intervertebral disc showed signal void with posterolateral herniation. Post-contrast images showed enhancement of the left posteroinferior T9 body and costovertebral joint	Expansion of the conus medullaris with abnormal T2 hyperintense focus. Enhancement in the epidural space at the level of T11 causing severe spinal canal stenosis with effacement of the thecal sac. T2 hyperintense signal with contrast enhancement within the T11 spinous process
Additional notes	No temporal association between spinal surgery and CPPD	Calcified ligamentum flavum, CT showed CLF + vacuum disc phenomenon at T10-T11	Specimen contained psammoma bodies, and meningeal perforation, not usually consistent with pseudogout	Biopsy of T11-T12 disc space, skip lesions, s/p lumbar fusion	Marked ossification of the ligamentum flavum bilaterally from T8 to T11 on CT	CT with bone algorithm was complementary to MRI and demonstrated ossified ligamentum flavum	Axial CT showed a calcified herniated disc. The foraminal location is unique	T9-T1O disk calcification on a conventional radiograph, the hypothesis is that CPP crystals migrated from the intervertebral disc toward the adjacent costovertebral joint through a radial tear of the annulus	

CPPD attacks may last up to months, unlike acute gouty attacks, which tend to be much briefer [1]. Our patient initially presented with symptoms after a respiratory infection and was treated with IVIG, which seemed to improve his condition. Treatment of spinal CPPD is often surgical with the addition of other therapeutics such as non-steroidal anti-inflammatory drugs (NSAIDS). A systematic review evaluating the current management strategies of CPPD states that there is limited evidence to support treatments such as the commonly used NSAIDS, colchicine and corticosteroids, and the less commonly used immunomodulators such as methotrexate, hydroxychloroquine, and biologics [4]. It is entirely possible that the initial episode was in fact AIDP, and the IVIG treatment helped keep it at bay. There has been no evidence found of CPPD flairs, especially in the spine, after an upper respiratory infection.

## Conclusions

Pseudogout causes calcium pyrophosphate deposition in the cartilage, typically affecting the appendicular skeleton. In rare cases, the spine, usually the cervical or lumbar areas, may be affected. The thoracic spine is the least commonly involved, which makes this case unique. There are very few cases of thoracic spine CPPD. Treatment with surgery is often indicated but patients may have long-lasting neurologic deficits.
