# Correction: A Case of Pseudogout Causing Thoracic Myelopathy

**DOI:** 10.7759/cureus.c329

**Published:** 2025-09-26

**Authors:** Emilee A Carpenter, Zaid Siddique, Ola El-Zammar, Adriana May, Kavya Mirchia

**Affiliations:** 1 Radiology, State University of New York Upstate Medical University, Syracuse, USA; 2 Pathology, State University of New York Upstate Medical University, Syracuse, USA; 3 Neuroradiology, State University of New York Upstate Medical University, Syracuse, USA

This article has been corrected to fix the following error in the Discussion section, first paragraph, second sentence: "Aging, previous joint injury, osteoarthritis, hyperparathyroidism, hypermagnesemia, hypothyroidism, and hemochromatosis are some of these risk factors [4,5]."

Hypomagnesemia, not hypermagnesemia, is associated with pseudogout. The above sentence has been corrected accordingly.

